# Core facets of divine forgiveness: a study across monotheistic religions

**DOI:** 10.3389/fpsyg.2025.1646554

**Published:** 2025-11-06

**Authors:** Francesca Giorgia Paleari, Francesca Vittoria Danioni, Valentina Valtulini, Daniela Barni, Aslı Bugay Sökmez, Sara Eissa, Yaakov Greenwald, Ariel Knafo-Noam, Camillo Regalia

**Affiliations:** 1 Department of Human and Social Sciences, University of Bergamo, Bergamo, Italy; 2 Family Studies and Research University Centre, Catholic University of Milan, Milan, Italy; 3 Department of Psychology, Cyprus Aydın University, Nicosia, Cyprus; 4 Department of Psychology, Catholic University of Milan, Milan, Italy; 5 Department of Psychology, The Hebrew University of Jerusalem, Jerusalem, Israel

**Keywords:** divine forgiveness, sin, monotheistic religions, cross-religion comparison, focus group, self-report

## Abstract

**Introduction:**

Among the religious factors that significantly contribute to believers’ well-being, research on the personal experience of divine forgiveness (DF) remains in its infancy. The aim of this study was to investigate similarities and differences in the conceptualization of DF, its conditional/unconditional nature, and the understanding of sin across the three main monotheistic religions.

**Methods:**

This was achieved by interviewing theologians (*N* = 3) through a focus group and having lay believers (*N* = 229, 63.8% female, *M*age = 33.09 years, SD = 13.81) from Christianity, Islam, and Judaism complete a self-report questionnaire.

**Results:**

The theologians’ and believers’ perspectives revealed that while there are shared aspects across religions (e.g., God’s mercy is greater than His justice), some differences are evident (e.g., the pathways to seek and achieve DF).

**Discussion:**

These findings make a significant contribution to the psychology of religion, shedding light on universal and culturally specific dimensions of this multidimensional phenomenon.

## 1 Introduction

The vast majority of the world’s population is affiliated with a religion (more than 85% in 2022 according to Statista, 2022) and will continue to identify with a religion in the future (Pew-Templeton Global Religious Futures project, 2022). According to the available literature on the topic, religiosity affects believers’ well-being with mixed results depending on the religious dimension considered. For example, Maltby and Day’s (2000) study found that, in both men and women, higher levels of depressive symptoms were associated with significantly higher scores on an extrinsic-social and extrinsic-personal orientations toward religion, and with significantly lower scores on intrinsic religious orientation. Independently of religious orientation, some religious behaviors like church attendance and meditative prayer are related to lower levels of depression and anxiety and higher levels of optimism, hope, self-esteem and life satisfaction, whereas some other behaviors like petitionary and ritualistic prayers were not significantly associated with them (Lambert et al., 2009; Maltby et al., 1999; Poloma and Gallup, 1991). Accordingly, as Pargament’s (1990, 1996) work has well documented, religion plays a key role in believers’ coping mechanisms designed to manage stress, some of which can have positive outcomes, some others negative (for a detailed discussion on the topic see James and Wells, 2003). Recent systematic review and meta-analysis studies concluded, however, that religious belonging, beliefs, and behaviors are predominantly associated with improved mental well-being and a reduced risk of mental disorders in English-speaking Christian or Jewish societies (Aggarwal et al., 2023; Braam and Koenig, 2019; Coelho-Júnior et al., 2022; Flannelly, 2017; Garssen et al., 2021; Yaden et al., 2022).

Among the religious factors that seem to play a key role in enhancing believers’ well-being, the personal experience of divine forgiveness may be a fundamental process for overcoming guilt, anxiety, and distress resulting from transgressions or sins committed, and many religious traditions have established procedures or contexts for believers to appeal to God for forgiveness. Nevertheless, despite its apparent relevance for psychological functioning and many religions worldwide, the role of personal experience of divine forgiveness remains largely unexplored. The few studies on the topic have been conducted almost exclusively in English-speaking Western countries with a Protestant Christian religious background. Within the scope of these studies, divine forgiveness has been recently defined as a “perceived absolution for a transgression or sin from a Supreme Being or Higher Power that is manifest in the individual’s cognition, affect, and/or behavior,” occurring “in relation to one’s sinful nature or in relation to individual transgressions/sins” (Fincham, 2022, p. 455). This definition closely aligns with that of human forgiveness, which has been conceptualized as a prosocial change in thoughts, feelings, or behaviors toward a perceived transgressor (McCullough, 2000). Being forgiven by the offended person and/or forgiving oneself may allow a positive change in the offender’s attitude toward the self and promote health (Carpenter et al., 2014; Pelucchi et al., 2017; Toussaint et al., 2017). Though receiving forgiveness after committing an offense may become the most relevant response to overcome the consequences of the negative action, forgiveness by God is distinct from human forgiveness in both its source and experiential implications (Fincham, 2022).

As already mentioned, research on divine forgiveness is quite limited, especially if compared to the body of literature concerning human forgiveness (McCullough et al., 2000; Woodyatt, 2017; Worthington and Wade, 2019). Since for believers, transgressions or offenses may be considered sins that compromise one’s relationship not only with other people, but also with God, the experience of divine forgiveness may have important implications for how people feel about themselves and behave toward others (Ludwig et al., 2025). For example, positive representations of God as kind and forgiving were associated with greater psychological well-being, whereas viewing Him as authoritative and vengeful was associated with greater distress and anxiety (Silton et al., 2014; Stulp et al., 2019). Accordingly, Fincham and May (2019) showed in samples of U. S. young adults that the perception of being forgiven by God was associated with fewer depressive symptoms among participants with lower levels of self-forgiveness. They also documented an inverse relationship between divine forgiveness and anxiety, as well as a positive association between divine forgiveness and life satisfaction (Fincham and May, 2024). Furthermore, other recent studies have confirmed the positive effect of divine forgiveness on general levels of well-being also considering the long-term effect by adopting longitudinal approaches (Chen et al., 2019; Long et al., 2020). Divine forgiveness has also been found to be positively and significantly correlated to future orientation (Kelliher Rabon et al., 2018), to directly associate with better levels of health-related social functioning (Svalina and Webb, 2012), to negatively correlate with substance use cravings and positively correlate with religiousness and spirituality (Skalski-Bednarz et al., 2024). It has been also recognized as a predictor of unconditional forgiveness of others (Krause and Ellison, 2003), in agreement with Huber et al. (2011), whose study has demonstrated that the experience of forgiveness by God is significantly and positively related to the tendency to forgive others, and research has also shown a strong positive association between divine forgiveness and self-forgiveness (Bassett et al., 2016; Krause and Ellison, 2003; McConnell and Dixon, 2012). Nonetheless, research has also reported conflicting or negative results. For instance, Ludwig et al. (2025) found that higher perceived divine forgiveness can increase self-forgiveness, which may, in some cases, reduce pro-relational behaviors like apologizing. Similarly, DeBono et al. (2017) showed that believing in a forgiving God may increase unethical behavior. Also, in some studies - for example, Krause and Ironson’s (2017) work and Toussaint et al. (2012) - researchers have identified negative personal health consequences of divine forgiveness.

According to the very recent Seeking-Experiencing Divine Forgiveness Model, the process of seeking God’s forgiveness after a sin committed begins as soon as the person has decided to pursue this kind of forgiveness after wrongdoing; specifically, a benevolent view of and a close relationship with God are positively associated to this search. Then, the likelihood of seeking divine forgiveness is related to experiencing receiving divine forgiveness over time (Fincham and Maranges, 2024). It has also been argued that among believers God representation may strongly vary; Sharp et al. (2021), in a review of aimed at considering the available measures devoted to assess this construct, have proposed a dual categorization of the concept, contrasting doctrinal representations (i.e., “God concept” or “head knowledge”) with experiential representations (i.e., “God image” or “heart knowledge”), which are likely to influence believers’ tendency toward divine forgiveness. Additionally, individuals who are characterized by an attachment to God which can be conceptualized as avoidant are overall less keen to seek for divine forgiveness because of their fear about the possibility of not obtaining it (Fincham and Maranges, 2025).

Accordingly, we argue that the process of asking and receiving divine forgiveness may depend on several factors and conditions. Belonging to a specific religious group is the least investigated aspect in the limited literature that has focused its attention on this topic. Nevertheless, doctrines, practices, and rituals regarding divine forgiveness show some aspects of similarity but also differences across monotheistic religions (Fincham, 2022); indeed, religions are relevant meaning systems for believers and are likely to affect how they conceptualize divine forgiveness and the conditions under which it occurs, together with the concept of sin (Tsang et al., 2005). Judaism, Christianity and Islam share a common origin, having emerged from the same spiritual lineage rooted in Abraham. Nurtured in the religious soil of the Middle East, they developed side by side. Yet, as they have matured, these closely related faiths now stand distinct from one another (Peters, 2018). In both Judaism and Christianity, for example, forgiveness is foundational to their doctrines, and believers are encouraged to forgive because God has forgiven them (e.g., Dorff, 1998; Van Oyen Witvliet, 2001). In Judaism forgiveness is mandatory if the transgressor has expressed repentance, compensated the victim, and committed to refrain from reiterating the offense by going through the process of teshuvah (“return”); in Christianity forgiveness is not conditional upon the transgressor’s repentance (Rye et al., 2000). Forgiveness from Allah is central also in Islam, which strongly encourages believers to grant it; however, differently from the Christian tradition, but like the Jewish one, forgiveness is not unconditional (Moucarry, 2004) but rather should be granted if the transgressor demonstrates repentance, apologizes, and explicitly begs for forgiveness (Mullet and Azar, 2009). Even though, according to all major monotheistic religions, sin is considered a central aspect of the human condition (Böttigheimer and Kamp, 2025), its nature and theological implications differ significantly among them. In Christianity, believers are regarded as sinners by nature, due to the doctrine of original sin inherited from Adam and Eve (Peters, 2018). In contrast, Islam does not recognize original sin; individuals are considered sinners by their actions. Similarly, both Judaism and Islam conceptualize sin primarily as a transgression of divine law.

These three Abrahamic religions have differentiated over time, and today it is well established that they follow distinct sacred texts (e.g., the Talmud for Jews, the New Testament for Christians, and the Qur’an for Muslims). These texts outline various rules, rituals, beliefs, and traditions, each with interesting nuances—for instance, *halakhah* (Jewish law), *canon law* (in Christianity), and *sharīʿa* (Islamic law); as well as distinct ritual practices and religious traditions such as Shabbat and the dietary laws in Judaism, the sacraments in Christianity, and the five pillars of Islam in the Muslim tradition. We may argue that these religions—along with the diverse cultures that may have intertwined with and influenced their later developments—offer distinct nuances regarding the rules that a believer is expected to follow, and the corresponding sins to be avoided. Likewise, they may differ in terms of the core religious practices to be observed, as well as in their conceptions of God, divine forgiveness, and sin, which may be understood as attainable through different means and under varying conditions.

Despite all these considerations, to the best of our knowledge the conceptualization of divine forgiveness and the conditions under which it is granted, together with the meaning of sin, have been empirically investigated very rarely among believers. Additionally, no study on divine forgiveness has simultaneously assessed the perspective of theologians and of lay believers. Considering both may help capture the complexity of the construct: as found for human forgiveness, scientists and common people may hold different yet complementary views of the topic (Kearns and Fincham, 2004). The current study aims at achieving these goals.

## 2 The present study

Based on all the above considerations and extending the previous and recent literature on this topic (i.e., Bartholomaeus et al., 2025), the main aim of the present study is to explore similarities and differences in the conceptualization of divine forgiveness, its conditional/unconditional nature, and of sin across the three main monotheistic religions by interviewing both theologians and lay believers belonging to Christianity, Islam, and Judaism. In particular, the current exploratory study aims to answer the following questions: What are the most common conceptualizations of divine forgiveness and sin among the experts and lay believers of the three main monotheistic religions? What are the most common beliefs about the conditions under which divine forgiveness occurs? Do these representations differ across monotheistic religions and, if so, in what way? Unlike the limited previous studies on the topic (Akl and Mullet, 2010; Bartholomaeus et al., 2025), this study addresses the above research questions by adopting a multi-method and multi-informant approach that includes the use of open-ended questions to both lay believers and theologians, in order to obtain a more comprehensive understanding of the phenomena investigated.

### 2.1 Methods

#### 2.1.1 Participants

Three senior theologians living in Italy took part in a 3-h focus group: 1 male Christian Catholic, 1 female Jewish, and 1 female Muslim theologian. Also, 229 lay believers (63.8% females), ranging in age from 18 to 77 (*M* = 33.09, SD = 13.81) and balanced across the three monotheistic religions considered (35.4% Christians, 32.7% Jews, 31.9% Muslims)^1^, completed an online anonymous questionnaire. Most of them were born in Italy (62.9%)^2^. Considering participants’ education level, most had completed secondary school (45.4%), an undergraduate degree (21%), or a Master’s degree (19.2%). Most participants were full time (41.9%) or part-time (11.4%) workers, while 38% were students. A small percentage were either in search of employment (1.7%), housewives (3.5%) or retired (3.5%).

The percentage of females was significantly lower in the Jewish sample (48%) than in the Christian (69.1%) or Muslim (74%) samples (*χ*^2^(4) = 14.61, *p* = 0.006). Moreover, the average age was significantly lower in the Muslim sample (*M* = 24.2, SD = 5.91) than in the Christian (*M* = 39.3, SD = 15.6) or Jewish (*M* = 35.0, SD = 12.92) samples (*F*(2,226) = 30.01, *p* < 0.001). Accordingly, Muslims had a lower education level than Jews which in turn were less educated than Christians (*χ*^2^(12) = 22.97, *p* = 0.028). Compared to the other two groups, there were fewer students and more retired among Christians, less working students among Jews, and less workers and retired, but more students among Muslims (*χ*^2^(12) = 46.86, *p* < 0.001).

On average, participants defined themselves as moderately religious (*M* = 2.61, SD = 0.98, range 1–4). Additionally, they reported to pray or participate in group religious rituals almost daily (*M* = 3.97, SD = 1.69, range 1–6), to know the doctrine of their religion quite well (*M* = 3.35, SD = 0.76, range 2–5), and to quite agree with it (*M* = 3.72, SD = 1.05, range 1–5). Compared to other two religious groups, Muslims reported to be significantly more religious, (*F*(2,226) = 23.96, *p* < 0.001, *ηp*^2^ = 0.18; *M*_Muslims_ = 3.15, *M*_Christians_ = 2.44, *M*_Jews_ = 2.27), to pray more (*F*(2,226) = 32.38, *p* < 0.001, *ηp*^2^ = 0.23; *M*_Muslims_ = 4.92, *M*_Christians_ = 3.83, *M*_Jews_ = 3.21), and agree more with the doctrine (*F*(2,226) = 62.78*, p* < 0.001, *ηp*^2^ = 0.36; *M*_Muslims_ = 4.58, *M*_Christians_ = 3.31, *M*_Jews_ = 3.35), when controlling for gender, age, education, and work condition.

#### 2.1.2 Material and procedure

The three theologians were recruited through judgmental sampling and invited to participate in a 3-h focus group, which was audio-recorded and transcribed. The focus group addressed the following thematic areas from each religious perspective: (a) the nature of divine forgiveness, (b) what sins are, (c) which are the conditions for being forgiven by God. These thematic areas were analyzed in terms of similarities and differences across the three religions (see Appendix 1 for the focus group guide in Supplementary material).

An anonymous self-report online questionnaire, including both open-ended and multiple-choice questions, was completed after providing informed consent by lay believers of the three monotheistic religions. Participants were informed about the main objectives of the study and were told that their participation was free and voluntary. The questionnaire included (a) socio demographic questions, (b) *ad hoc* questions aimed at measuring the religious involvement, (c) ad hoc questions aimed at assessing what divine forgiveness is (participants were asked to report three keywords associated with divine forgiveness), (d) ad hoc questions aimed at assessing what sins are (open ended questions thereafter codified into categories by two independent researchers), and (e) which are the conditions under which divine forgiveness occurs (open ended questions thereafter codified into categories by two independent researchers; see Appendix 2 in Supplementary material for more details).

The study was approved by the Ethical Committee of the Department of Psychology of the Università Cattolica del Sacro Cuore of Milan, Italy (Protocol number: 137/24) and followed the APA standard ethical guidelines for research.

#### 2.1.3 Data analysis

Qualitative data from theologians were analyzed in relation to each of the proposed thematic areas, using a combination of thematic and content analysis. The thematic areas mentioned in the previous section were analyzed in terms of similarities and differences across the three religions (see Appendix 1 for the focus group guide in Supplementary material). The first step in analyzing the focus group was to transcribe the discussion taking place in it. Member checking – a technique in which data or results are returned to participants to check their accuracy – was also undertaken to increase rigor (Bowen, 2005): its results and the focus group transcript constitute the qualitative data from theologians that were analyzed. A non-literal transcription and a paper-and-pencil thematic analysis of the material were employed. Some elements of non-verbal behavior, such as facial expressions, gestures, head nodding, were also taken into account; for example, when we did not receive response from every theologian, we made sure that expert(s) who did not provide a verbal response agreed with and supported the views of those who spoke before them, for instance through nods, smiles, or approving looks.

Quantitative data from lay believers were statistically analyzed through SPSS. An analysis of frequencies was carried out to detect the most reported keywords (each participant provided 3 keywords) associated with divine forgiveness; two authors independently analyzed the reported keywords and uniquely coded them using the noun; for example, the words “forgive” and “forgiven” were coded as “forgiveness.” In case of disagreement a third author was involved; interrater agreement was found to be excellent (Cohen’s Kappa = 0.96). Responses to an open-ended question concerning the nature of sin – what is a sin? – were coded into the following four categories proposed by two of the authors after an initial review of participants’ responses and grounded in prior literature on moral and religious cognition: (1) a harm committed toward the self or others^3^ (41% of respondents), (2) the violation of religious or moral laws (42.8% of respondents), (3) a distancing from God (18.8% of respondents), (4) something wrong or bad (17% of respondents). The categories were developed through an inductive process, based on participants’ textual open-ended responses, rather than imposed *a priori*. As such, the categories emerged from a careful reading of participants’ own language and formulations. In case of disagreement a third author was involved; interrater agreement was found to be good (Cohen’s Kappa = 0.73). Responses that included two or more categories were counted in all relevant categories. When investigating the conditions under which divine forgiveness occurs, participants indicated whether God never fully forgives (11.8%), whether’ God’s forgiveness is unconditional (52.8%) or whether God forgives only under certain conditions (35.4%); only respondents who selected this last option were asked to report conditions under which divine forgiveness occurs using an open-ended question. Afterwards, responses were coded into nine categories proposed by two of the authors, following the same procedure described above. The following categories emerged: sincerely repenting (19.7%), personally improving (8.7%), committing to not repeat the wrongdoing (6.1%), asking for forgiveness (4.4%), making amends (4.4%), acknowledging a wrongdoing (3.1%), praying (1.7%), having committed a minor sin (0.9%), confessing or adopting other rituals (0.9%). In case of disagreement a third author was involved; interrater agreement was found to be excellent (Cohen’s Kappa = 0.87). Responses that included two or more categories were counted in all relevant categories.

To understand whether the conceptualization of sin and divine forgiveness, as well as the conditions under which divine forgiveness was experienced, was associated with participants’ religion when controlling for their significantly different socio-demographics (i.e., gender, age, education, and work condition), we carried out binomial logistic regressions.

## 3 Results

### 3.1 Theologians’ focus group

Results are organized based on similarities and differences across religions with regard to the thematic areas considered (the nature of divine forgiveness, what sins are, which are the conditions for being forgiven by God). In Table 1 we report the main results of the study.

**Table 1:** Main results of the focus group.

Thematic area	Religious comparison
The nature of divine forgiveness	
DF is one of the fundamental aspects of God and it is always available for everyone.	C ≅ M ≅ J
God’s mercy is greater than his justice.	C ≅ M ≅ J
Ways through which DF is sought:	
DF is an act mediated by a specific rite and a specific religious figure.	C
DF occurs through a direct relationship with God, without the need for an intermediary.	M ≅ J
Practices and rituals for divine forgiveness:	
The Sacrament of Confession.	C
Yom Kippur.	J
Ramadan.	M
What sins are	
Sin is a transgression that goes against God’s will.	C ≅ M ≅ J
DF for sins against others vs sins against God:	
Sin against others: to receive God’s forgiveness, Muslims and Jews must first seek forgiveness from the offended individual before seeking divine forgiveness.	M ≅ J
Seeking interpersonal forgiveness is not mandatory to get God’s forgiveness.	C
The original sin:	
The first human sin in order to learn to exercise discernment.	J
The first human sin, that is Adam’s non-firmness, his failure to safeguard human purity.	M
The man’s claim to decide what is good and what is evil overriding God’s commandment.	C
Conditions for divine forgiveness	
Conditions require a personal commitment in the domain of thoughts, intentions, and actions, which must be directed toward the good.	C ≅ M ≅ J
Key concepts emerged:	
Willingness to renew relationships, reconciliation with the offended person and the community, repentance, and *teshuvah*.	J
Taking responsibility for one’s actions, undergoing a transformation in life for the better, a ‘pain of/for sins’ that invokes God’s forgiveness without pretending it.	C
Willingness to be merciful toward the other, sincere feeling of repentance without vengeance and hatred, acting to repair the harm done, and *tawba*.	M
Guarantee of being forgiven.	
The words of absolution pronounced by the religious figure during the Sacrament of Confession guarantees that the believer has been forgiven by God.	C
There is no external authority that guarantees DF.	M ≅ J

C, Christians; M, Muslims; J, Jews. ≅ imply a similarity among religions.

#### 3.1.1 The nature of divine forgiveness

According to all three theologians, regardless of their religious affiliation, divine forgiveness is one of the fundamental aspects of God and it is always available for everyone. They all emphasised that “*Divine forgiveness is one of the fundamental aspects of (God’s) unconditional love, which also includes merc*y. *[…] Forgiveness is present and is absolutely unconditional and infinitely a thousand times greater than resentment*.” In fact, God’s mercy is greater than His justice and human beings must put themselves in the position to open up and accept the gift of divine forgiveness. The theologians stated in agreement that *“Mercy and justice are two attributes of God, […] With one detail, however: the relationship between these two attributes is overwhelmingly in favor of mercy […] God’s mercy is a thousand times greater than his justice. […] One can say ‘I forgive you’, but it can be totally ineffective […] And on what does it depend? Not on the fact that God has not fully accomplished his mercy, but that man has not yet fully accepted it.”*

According to all theologians, God forgives and also recommends forgiveness among the believers and advises not to take revenge and to do good to others: both self-forgiveness and interpersonal human forgiveness should be inspired by divine forgiveness. Indeed, the theologians declared in unison that *“He presents Himself as the one who forgives and recommends it to human beings […] God advises forgiveness to human beings at all relationship levels (between groups and between individuals) and recommends not only to forgive but also to respond to evil with good […].”*

From the discussion and opinions of the theologians during the focus group, a primary difference across religions concerns the way divine forgiveness is sought: for Catholic Christians, forgiveness is an act mediated by a specific rite (the Sacrament of Confession) and a specific religious figure (typically a priest), whereas for Jews and Muslims, it occurs through a direct relationship with God, without the need for an intermediary. As reported by the Christian theologian: *“The word of absolution is performative: it produces what it declares. If Christ obtained forgiveness of sins once and for all for everyone, the positive action of the Sacrament (of Confession) guarantees that forgiveness for the believer.”* As claimed by the Muslim and Jewish theologians instead: *“The non-need […] to be forgiven through man […] gives each individual the possibility to ask for forgiveness directly, within himself and with his God. Therefore […] removing the figure of the mediator has tried to give the individual such an importance to get directly in touch with his own spirit, within himself and with God […].”*

As a result, practices and rituals for divine forgiveness also differ among the three religions. As mentioned above, Christianity has the Sacrament of Confession, while Judaism and Islam do not, but each has specific solemn moments for seeking divine forgiveness such as Yom Kippur in Judaism and Ramadan in Islam. In Christian tradition, the Sacrament of Confession is one of the seven sacraments of the Catholic and Orthodox Churches during which believers individually confess their sins to a religious figure who offers forgiveness in the name of God. As the Christian theologian declared: *“When the believers profess themselves to be sinners and implore God’s forgiveness (during the Sacrament of Confession) […] the believers receive forgiveness from God’s mercy […] and together they are reconciled with the Church.”* In Jewish tradition, Yom Kippur is the holiest celebration, which is dedicated to prayer and fasting to atone for the sins of the past year: *“With the Religious New Year […] begins the ‘ten days’ in which one must examine his/her actions […] then one must reconcile with all with whom the relationship has broken down […] At the end of the ten days we celebrate the Day of Kippur, during which we pray and fast communally […] The liturgy ends with the sounding of the Shofar announcing divine forgiveness.”* In Islamic tradition, instead: *“An example of a solemn period is the period of Ramadan, when people fast and there are community moments […] On this occasion, the gates of Paradise are open, so it is also a time for self-forgiveness and collective forgiveness.”* Ramadan is one of the Five Pillars of Islam, the holy month of fasting commemorating the first revelation of the Quran. At the end of the month, Eid al-Fitr, the feast of breaking the fast, is celebrated.

#### 3.1.2 What sins are

During the focus group discussion, theologians mainly focused on the differences in the definition of Original Sin across the three religions, without clearly emphasizing the similarities, which were taken for granted. However, based on the responses obtained through the member checking method and the fruitful dialogue on the definition of Original Sin, it can be affirmed that the theologians implicitly agreed that sin can generally be defined as a transgression (whether in thought, behavior committed or omitted, etc.) that goes against God’s will.

The main difference among the religions that emerged is, as mentioned, the theme of Original Sin. From the Jewish perspective, the “first human sin” is seen as a necessary wrongdoing in order to learn to exercise discernment. In fact, as the Jewish theologian explained: *“The ‘first human sin’ is a necessary wrong in order to learn to distinguish between good and evil (which is why Judaism does not speak of ‘Original Sin’) […] However, the moment a person learns to exercise discernment, sin constitutes a transgression with respect to the revealed divine teachings […].”* According to Islam, on the other hand, human beings are born pure and must safeguard the beauty within themselves (“fitrah”), so the ‘first human sin’ is seen as a failure to maintain this purity by transgressing divine instructions. The Muslim theologian declared: *“The real sin is non-firmness. […] Then (Adam/the human being) is born pure, he has no burden on his soul. He is born pure, the only path he has to follow is to guard the beauty he has… and the very key word is taqwa. Taqwa is really ‘to guard’: the real effort of the believer.”* Differently, for Catholic Christians, the Original Sin consists of the humanity’s claim to decide what is good and what is evil, stemming from a lack of faith and disobedience to God’s commandment. As the Christian theologian said: *“What does (Original) sin mean? […] It means that […] I do not accept that someone else (God) disposes what is good and what is evil… but I want to be an actor in this decision. […] If so, that fall (the Original sin) means precisely man’s claim to be God.”* Finally, in Judaism and Islam a significant distinction between sins against others and against God emerges, whereas in Christianity does not. In fact, the Jewish and Muslim theologians stated that: *“A distinction is made between the rights of God and the rights of people over the believer. […] In the case of an offense against others, one must seek forgiveness from the offended person*.” In other words, for Muslims and Jews, but not for Catholic Christians, seeking forgiveness from God is not enough when one commits a sin against others. Muslims and Jews must first apologize and seek forgiveness from the offended individual before seeking divine forgiveness. In Christian tradition, instead, seeking interpersonal forgiveness is encouraged and recommended but not mandatory to get forgiveness from God.

#### 3.1.3 Conditions for divine forgiveness

In all three religions, the conditions for being forgiven by God require a personal commitment not only in the domain of thoughts and intentions (e.g., taking responsibility, willingness to renew relationships, intention to follow God’s example, and be merciful to others), but also actions (e.g., reconciling and renewing relationships, transforming behavior or life for the better, acting to repair the harm done). Thoughts, intentions, and actions must be directed toward the good. In Judaism, conditions for being forgiven by God are the willingness to renew relationships (intentions), reconciliation with the offended person and the community, and renewal of relationships (action), while a change in feeling is not essential. Moreover, “teshuvah” (repentance and return to God through reconciliation with Him and others) is fundamental to being forgiven. This concept is closely connected to personal faith and one’s relationship with God and others. There is no external authority that guarantees divine forgiveness. As the Jewish theologian affirmed: “*In order to experience God’s forgiveness in this kind of relationship, if justice is historical it must also pass through human forgiveness. Let us take an example, if I do not pray… it is a matter between me and God, and I will have to ask God for forgiveness […] But if I offend someone it is not enough that I repent in order to receive divine forgiveness […] I must first be reconciled with them […] otherwise God’s love is there but cannot become active. […] God’s Chesed (loving kindness and grace) is unconditional but in order to manifest itself in history it needs the human contribution that is available to renew relationships.”*

For Christianity, the conditions for receiving divine forgiveness include taking responsibility for one’s actions (thoughts) and undergoing a transformation or change in behavior or life for the better (action). The ‘pain of/for sins’ (feelings) indicates the necessary humility of the believer who invokes God’s forgiveness without pretending it. The words of absolution pronounced by the religious figure during the Sacrament of Confession guarantees that the believer has been forgiven by God. In the words of the Christian theologian: *“Does God forgive everything and always infinitely? Yes, but the problem is how this involves the freedom of man who opens himself to that gift as a gift […] The word of a Sacrament is performative […] For Christianity […] a person (the religious figure) is the Sacrament of God […] That is, in that person God is fully manifested: hence, in a concrete, detailed, circumstantial man […] So the real problem is not in God but in the extent to which man makes God’s work his own, and it is here that man’s responsibility is significantly involved, that is to let God do His work of justification (=making human fair/just) in him […] within a process of purification […] of new life.”*

In Islam, the conditions for being forgiven are a willingness to be merciful toward the other (thought), sincere repentance without vengeance and hatred (feeling), acting to repair the harm done (action). Allah in the Quran defines himself as the One who forgives those who perform “tawba” (returning repentantly to God). So, this is related to one’s relationship and image with God. Forgiveness, both given and received, affects the believer, allowing them to experience joy and salvation. In this regard, the Muslim theologian argued: *“So here the Qur’an shows […] three levels of forgiveness. Ta′fuw is […] do not seek revenge, […] ‘tasfahu’ means ‘turn over a new page’ […] do not always return to remember […] then ‘taghfiru’, just ‘cover it up’. […] There is a part of (divine) forgiveness that […] has the condition that you need to be forgiven by the offended person […] There is God’s right and man’s right […] when you have sinned towards your neighbour, it is your neighbour who must forgive you […] ‘What should I do when I have to respond to an evil?’. It (the Quran) says: ‘You respond to that evil not with an evil in the same measure, but you respond with a good to that evil […].”*

### 3.2 Lay believers

#### 3.2.1 Keywords associated with divine forgiveness

In Table 2 we report the main findings of the study with lay believers. Participants were asked to freely report three keywords associated to the concept of divine forgiveness and the most cited ones are the following: Mercy (30.1%), Love (21.4%), Repentance (14.8%), Peace (9.2%), God (9.2%), Forgiveness (7.9%), Compassion (7%), Grace (5.2%), Sin (4.8%), and finally Redemption (3.9%). Although the overall binomial logistic regression models were not always significant, results suggested that religious groups significantly differed with respect to the likelihood of reporting the following terms: Mercy [*χ*^2^(16) = 66.47; *p* < 0.001; Nagelkerke *R*^2^ = 0.36; cases correctly classified = 77.7%], Love [*χ*^2^(16) = 18.43; *p* = 0.300; Nagelkerke *R*^2^ = 0.12; cases correctly classified = 78.6%]; Repentance [*χ*^2^(16) = 31.61; *p* = 0.011; Nagelkerke *R*^2^ = 0.23; cases correctly classified = 86.9%], Peace [*χ*^2^(16) = 36.20; *p* = 0.003; Nagelkerke *R*^2^ = 0.32; cases correctly classified = 91.7%], God [*χ*^2^(16) = 30.05; *p* = 0.018; Nagelkerke *R*^2^ = 0.27; cases correctly classified = 91.3%], Compassion [*χ*^2^(16) = 25.59; *p* = 0.060; Nagelkerke *R*^2^ = 0.27; cases correctly classified = 94.8%], and Sin [*χ*^2^(16) = 15.61; *p* = 0.480; Nagelkerke *R*^2^ = 0.21; cases correctly classified = 95.2%].

**Table 2:** Main findings of the study with lay believers.

Keywords for DF	Results	Nature of sins	Results	Conditions of DF	Results
Mercy	M > C > J	Violation of religious or moral laws	M > J > C	Sincere Repentance	M > C and J
Love	C > M	A harm committed toward the self or others	C > J > M	Commitment to not repeat the wrongdoing	M > C and J
Repentance	M > C and J	A distancing from God	M and C > J	Personal improvement	J > C
Peace	M > C and J	Something wrong or bad	C = J = M	Asking for forgiveness	C = J = M
God	J > C and M			Wrongdoing acknowledgment	C = J = M
Compassion	M > J > C			Praying	C = J = M
Sin	J > M			Making amends	C = J = M
Forgiveness	C = J = M			Committing minor sin	C = J = M
Grace	C = J = M			Confession or other rituals	C = J = M
Redemption	C = J = M				

C, Christians; M, Muslims; J, Jews.

Specifically, Muslims were 3.91 times more likely to report the keyword Mercy than Christians (Wald = 8.81, *p* = 0.003), who in turn were 5.61 times more likely than Jews (Wald = 18.18, *p* < 0.001). Muslims were also 8.00 and 7.08 times more likely to report the word Repentance (Wald = 11.01, *p* < 0.001; Wald = 10.17, *p* = 0.001), and 24.42 and 14.48 times more likely to report the word Peace (Wald = 8.30, *p* = 0.004; Wald = 7.15, *p* = 0.007) than Christians and Jews, respectively. Jews were 3.53 and 10.45 times more likely to indicate the keyword God than Christians and Muslims, respectively (Wald = 3.99, *p* = 0.046; Wald = 9.07, *p* = 0.003). Christians were 3.12 times more likely to report the word Love (Wald = 4.84, *p* = 0.028) than Muslims. Muslims were 22.05 times and Jews were 12.32 times more likely to report the keyword Compassion (Wald = 5.74, *p* = 0.017; Wald 4.32, *p* = 0.038) than Christians. Jews were 10.33 times more likely to report the keyword Sin (Wald = 4.14, *p* = 0.042) than Muslims^4^.

For the others (Forgiveness [*χ*^2^(16) = 13.12; *p* = 0.664; Nagelkerke *R*^2^ = 0.13; cases correctly classified = 92.1%], Grace, [*χ*^2^(16) = 6.82; *p* = 0.977; Nagelkerke *R*^2^ = 0.09; cases correctly classified = 94.8%], and Redemption, [*χ*^2^(16) = 25.17; *p* = 0.067; Nagelkerke *R*^2^ = 0.37; cases correctly classified = 96.9%]), no statistically significant differences emerged, as suggested by the group comparisons.

#### 3.2.2 Nature of sin

Results suggested that religious groups significantly differed with respect to the likelihood of conceptualizing sins as follows: violation of religious or moral laws [*χ*^2^(16) = 50.95; *p* < 0.001; Nagelkerke *R*^2^ = 0.27; cases correctly classified = 69%], an harm committed toward the self or others [*χ*^2^(16) = 31.15; *p* < 0.013; Nagelkerke *R*^2^ = 0.17; cases correctly classified = 63.3%], a distancing from God [*χ*^2^(16) = 18.29; *p* = 0.307; Nagelkerke *R*^2^ = 0.12; cases correctly classified = 80.8%]. Specifically, Muslims were 9.65 times (Wald = 25.00, *p* <. 001) and Jews were 4.24 times (Wald = 13.03, *p* <. 001) more likely than Christians to conceptualize sin as a violation of religious or moral laws. Christians were 4.44 times (Wald = 12.60, *p* < 0.001) and Jews were 3.57 times (Wald = 9.08, *p* < 0.001) more likely than Muslims to report sin as a harm committed toward the self or others. Muslims were 3.54 times (Wald = 5.12, *p* = 0.024) and Christians were 2.99 times (Wald = 4.54, *p* = 0.033) more likely than Jews to report sin as distancing from God.

No statistically significant differences emerged based on participants’ religions in the classification of sin as something wrong or bad [*χ*^2^(16) = 13.71; *p* = 0.920; Nagelkerke *R*^2^ = 0.10; cases correctly classified = 83.4%].^5^

#### 3.2.3 Conditions of divine forgiveness

When considering the conditions under which divine forgiveness occurs, although the overall binomial logistic regression models were not significant, results suggested that religious groups were significantly different with respect to the likelihood of mentioning the following conditions or circumstances for divine forgiveness to occur: sincerely repenting [*χ*^2^(16) = 20.74; *p* = 0.189; Nagelkerke *R*^2^ = 0.14; cases correctly classified = 80.8%], committing to not repeat the wrongdoing [*χ*^2^(16) = 21.63; *p* = 0.155; Nagelkerke *R*^2^ = 0.24; cases correctly classified = 93.9%] and personally improving [*χ*^2^(16) = 19.37; *p* = 0.250; Nagelkerke *R*^2^ = 0.18; cases correctly classified = 91.3%]. For the other conditions (asking for forgiveness [*χ*^2^(16) = 20.56; *p* = 0.197; Nagelkerke *R*^2^ = 0.28; cases correctly classified = 96.1%], acknowledging a wrongdoing [*χ*^2^(16) = 16.58; *p* = 0.413; Nagelkerke *R*^2^ = 0.29; cases correctly classified = 96.9%], praying [*χ*^2^(16) = 26.17; *p* = 0.052; Nagelkerke *R*^2^ = 0.70; cases correctly classified = 97.8%], making amends [*χ*^2^(16) = 17.30; *p* = 0.324; Nagelkerke *R*^2^ = 0.22; cases correctly classified = 94.8%], committing minor sin [*χ*^2^(16) = 17.68; *p* = 0.343; Nagelkerke *R*^2^ = 0.78; cases correctly classified = 99.1%], confessing or adopting other rituals[*χ*^2^(16) = 17.89; *p* = 0.330; Nagelkerke *R*^2^ = 0.79; cases correctly classified = 99.6%]), no statistically significant differences emerged, as also suggested by the group comparisons.

Specifically, Muslims were 3.99 and 2.85 times more likely than Christians and Jews (Wald = 7.29, *p* = 0.007; Wald = 4.73, *p* = 0.030) to report sincerely repenting as a condition to be forgiven by God. Also, Muslims were 10.83 times and 8.60 times more likely than Christians and Jews (Wald = 6.40, *p* = 0.011; Wald = 5.75, *p* = 0.017) to report committing to not repeat the wrongdoing as a condition for divine forgiveness. Jews were 7.79 times more likely than Christians (Wald = 6.56, *p* = 0.010) to indicate personally improving as a condition for divine forgiveness^6^.

## 4 Discussion and conclusion

The study of divine forgiveness has recently gained the attention of scholars, thus determining the increase of scientific literature specifically dealing with this topic (see for example the very recent works of Fincham, 2022; Fincham and Maranges, 2024, 2025). Indeed, when a person has committed a wrongdoing, together with the benefits derived from self-forgiveness and interpersonal forgiveness of the victim of the offense, divine forgiveness may be extremely beneficial. The benefits of divine forgiveness mainly deal, according to the limited literature on the topic, with its capability to affect lay believers’ well-being (Fincham and May, 2019) also after a considerable amount of time (Chen et al., 2019; Long et al., 2020).

Research on divine forgiveness is in its infancy, but recent studies have highlighted how this may be a religious factor likely to play a key role in enhancing believers’ well-being. Nevertheless, the personal experience of divine forgiveness remains largely unexplored and there is very little empirical evidence on the conceptualization people have of divine forgiveness, sin, and the conditions under which it occurs. However, to generate an empirically based definition of the construct it is essential to examine how believers from different religions, whether experts or laypeople, understand these important religion-related constructs. As shown by previous research on the topic, similarly to human forgiveness (McCullough, 2000; Paleari et al., 2009; Subkoviak et al., 1995; Woodyatt and Wenzel, 2013), this “perceived absolution” may be inferred by the presence of positive thoughts, feelings, and/or experiences related to God (e.g., a sense of peace in the relationship with God), but also by the absence of negative ones, similarly to what scientific literature on interpersonal and self-forgiveness suggests. Indeed, when lay believers commit sins, divine forgiveness is not felt as an immediate consequence, since God may be perceived as distant or even avoidant as a consequence of the wrongdoing committed (DeBono et al., 2017; Kim et al., 2022). Additionally, divine forgiveness can both hinder and encourage constructive responses to conflict; Indeed, in Ludwig et al.’s work (2025) DF predicted a lower likelihood of apologizing due to self-forgiveness, showing that, in some cases, DF may reduce responsibility-taking. However, their study also showed that divine forgiveness increased feelings of gratitude and humility. Thus, DF plays a dual role: it may reduce interpersonal accountability under certain psychological pathways (via self-forgiveness), while under others it may promote reconciliation and prosocial repair (via gratitude and humility).

In order to capture the complex nature of divine forgiveness across different religions, we developed two different studies, the first qualitative involving three senior theologians belonging to three main monotheistic religions, the second quali-quantitative aimed at gathering data from lay Christian, Jewish, and Muslims believers. Using a different approach in each study, we investigated if the meaning assigned to sin and divine forgiveness changes across monotheistic religions and if so under which respect. This allows us to disentangle the complexity that we suppose lies within the concept of divine forgiveness, thus also considering, for the first time in the literature in this topic, the perspective of both theologians and believers.

Examining how believers from different religions, whether experts or laypeople, understand divine forgiveness is important for several reasons. First, it is crucial to generate an empirically based definition of the construct upon which to develop measurement instruments capable of capturing it in all its facets, as suggested by Kearns and Fincham (2004) regarding human forgiveness. The research conducted so far on experienced divine forgiveness has used simple measures (composed of one or two items; Harris et al., 2008; Toussaint et al., 2001), almost always unidimensional (Fincham and May, 2019; Kim et al., 2022), which therefore do not fully allow to capture the complex and presumably multidimensional nature of the concept. Trying to address these limitations, using a self-report questionnaire, Akl and Mullet (2010) found that French Christians conceptualize God’s forgiveness as unconditional, sensitive to circumstances in which offenses are committed, but also as sometimes absent due to God’s lasting resentment. Very recently, Bartholomaeus et al. (2025), in analyzing the psychological experience of divine forgiveness across monotheistic religions, have found a prototypical structure of divine forgiveness characterized by central (“Comes from God”) and peripherical (“Absolution/sin is removed”) traits. Despite being intriguing, these aspects do not seem to be fully represented in the available measures designed to assess divine forgiveness as experienced by believers.

Second, individuals’ conception of divine forgiveness is likely to influence their proneness to seek it. For example, the idea that divine forgiveness is granted unconditionally and easily, or conversely, only if specific and potentially difficult conditions are met (e.g., refraining from committing the same sin again), may influence how and the extent to which individuals seek it. Therefore, to understand how people navigate the process of forgiveness, it may be important to examine how they conceptualize it.

Third, understanding believers’ conceptions of divine forgiveness can enhance spiritual practices and interventions. By exploring how believers view and experience divine forgiveness, religious leaders can address misconceptions and improve spiritual guidance, helping individuals overcome fears or misunderstandings about seeking forgiveness and its positive outcomes.

Fourth, a better understanding of this construct may also help explore, prevent and counteract its possible negative implications at various levels - intrapersonal, interpersonal, and societal. In fact, perceiving oneself as forgiven by God may diminish personal responsibility (Ludwig et al., 2025) and foster negative personal health consequences (Krause and Ironson, 2017; Toussaint et al., 2012). Furthermore, it may also exacerbate feelings of guilt or distress under conditions of religious doubt, rather than alleviating them, leading to lower life satisfaction or increased depressive symptoms (Upenieks et al., 2023). Additionally, DF might strain relationships when harmed parties expect apology and repair, but the transgressor, relying on perceived DF, bypasses human reconciliation (Saleem and Sitwat, 2025).

A complex pattern of conceptualization of sin and divine forgiveness across the three religions emerged from the theologian interviews, indicating both cross-religion similarities, in line with previous research (Bartholomaeus et al., 2025), and differences. Based on the theologians’ perspectives, differences mainly emerge in the “practical” aspects of divine forgiveness rather than the theoretical ones. Indeed, while the nature and the essence of divine forgiveness are shared among religions, the ways in which believers seek divine forgiveness, as well as the rituals and practices they adopt, differ. For example, while Christians recognize the role of a spiritual guide (the priest) and a Sacrament (Confession) to obtain divine forgiveness, for the other religious groups it involves a more direct relationship with God and more personal reflection. While the conceptualization of sin does not vary according to the theologians’ religions (it is a transgression that goes against God’s will), the Muslim and Jewish theologians emphasized the relevance of distinguishing between sins against other people and sins against God. Indeed, Christians emphasize mutual forgiveness as part of the relationship with God: accordingly, in the Lord’s Prayer they say “And forgive us our trespasses, as we forgive them that trespass against us,” indicating that divine forgiveness is linked to human forgiveness. In contrast, for Jews, divine forgiveness is contingent upon seeking forgiveness from the victim when the transgression is also interpersonal in nature.

All theologians agree on the conditional nature of divine forgiveness, which implies a personal commitment in the domain of thoughts, intentions, and actions directed toward the good. However, theologians report different conditions for divine forgiveness across religions (e.g., a genuine effort to reconcile with the offended person and doing ‘teshuvah’ for Jews, taking responsibility for one’s own actions and confessing sins to a priest while invoking God’s forgiveness with humility for a new life for Christians, and acting to repair the evil done or received with a good and doing ‘tawba’ for Muslims), with only Christians being really assured of being forgiven through the Sacrament of Confession.

With regard to the nature of divine forgiveness according to lay believers, it is worth noting a heterogeneous representation of this concept especially for Muslim respondents; indeed, they report several keywords as more frequent compared to the other religious groups (Mercy, Repentance, Peace, and Compassion). Interestingly, Mercy appears to be a core concept across informants (theologians and believers). In contrast, the most commonly mentioned word among Christians is Love, while Jews associate the concept of divine forgiveness not only to the concept of Sin but also to the concept of God Himself, thus emphasizing the direct personal relationship with God promoted in this religion. Sin is conceptualized differently across religions, with the greatest difference in meaning probably emerging between Muslims, who mainly view sins as transgressions against God and His laws, and Christians, who instead hold a more relational perspective of sin. Finally, it is interesting to note that Repentance, which was not highly stressed by all theologians, emerges as a core concept of divine forgiveness among lay believers. Indeed, Repentance is not only a key trait of divine forgiveness, being the third most mentioned keyword, but also the main cited condition for obtaining divine forgiveness across the three religions (although cited more frequently by Muslims, in line with recent literature; see, for example, Saleem and Sitwat, 2025). Additionally, while Christians report a mostly unconditional perception of divine forgiveness, Muslims perceive the importance of a Commitment to not repeat the wrongdoing - indeed, Allah is Al-Qahhar (The Subduer) and Al-Muntaqim (The Avenger) punishes persistent sinners (Saleem and Sitwat, 2025) -, and Jews report the need for Personal improvement in order to grant divine forgiveness - in fact, for example, *teshuvah* should be seen as a full-blown return to the right path and to good standing with community and God (Dorff, 1998). The unconditional-conditional nature of divine forgiveness is a quite interesting and controversial topic that is now under the lenses of researchers working in this area. In agreement with Fincham and Maranges (2024), it is worth noting that across the three major monotheistic religions - but also across theologians and lay believers - beliefs about the conditional nature of divine forgiveness vary considerably. Different views are not shaped solely by religious teachings; rather, they are also informed by cultural, familial, and social influences, and by how people view and relate to the deity. All these factors may contribute to a wide array of interpretations: understanding whether divine forgiveness is perceived as conditional - and which conditions are the most important in each religion and for each individual - is crucial, as this perception significantly affects whether and how individuals seek forgiveness from God (Fincham and Maranges, 2024). It is also important to note that the results of the study carried out with lay believers remained largely consistent in terms of meaning when excluding or including the covariates in the analyses, with only small variations observed. Specifically, when covariates (gender, age, education, and work condition) are not included in the analyses, some differences (e.g., with regard to the keywords and to the conditions associated with divine forgiveness) between religious groups may go unnoticed, whereas they emerge when these covariates are instead considered. These results suggest that controlling the role of sociodemographic variables in the study of divine forgiveness can lead to a more nuanced and complex understanding of lay believers’ reports of keywords and conditions associated with divine forgiveness since the differences observed cannot be attributed solely to religious affiliation, but also to other personal characteristics.

Every study has some weaknesses, and this is, of course, no exception. In fact, we must mention that the sample was Italian and of convenience-based for Christian and Muslim participants, while data from Jews were primarily collected in Israel, among English-speaking believers, through Prolific (see footnote 2 for more details on this). Also, the focus group presented involved only three theologians, each one working in Italy and belonging to a specific religious denomination (e.g., the Christian theologian was Catholic); this, in turn, could have influenced their responses.

Our coding approach, based on inductive content analysis and informed by prior literature, allowed us to capture participants’ language while ensuring comparability across responses. This method helped preserve semantic specificity and reflect subjective understandings of sin. However, the process was not without limitations: Ambiguous responses sometimes required merging conceptually distinct categories. Furthermore, the analytic structure may have limited the identification of more unexpected or nuanced themes. Regarding the reliance on open-ended keyword responses, instead, it may reflect immediate associations rather than deeply held conceptualizations. Moreover, it is important to acknowledge that the categorization and qualitative analysis of responses to the open-ended questions may have been subject to interpretation biases. However, in order to mitigate this risk, independent coders from different religious backgrounds (one Muslim and one Christian) were selected, inter-rater reliability (Cohen’s Kappa) was calculated, and any uncertainties or ambiguities were discussed and resolved collaboratively with all members of the research team involved in the study. Regarding the conditions of divine forgiveness, the sample of lay believers was cut by one third. This limitation may have impacted the diversity and representativeness of the viewpoints collected. Therefore, findings on this specific aspect should be interpreted with caution. Further research should also take into account the extent to which believers actually feel they understand the doctrine of their religion.

However, the study has the great merit of contributing to a relevant topic in scientific research, namely the conceptualization of divine forgiveness. The limited research available on divine forgiveness has clearly shown its positive effects in terms of well-being (Chen et al., 2019; Fincham and May, 2019; Long et al., 2020). However, in order to develop a more comprehensive understanding of its effects, it is crucial to gain knowledge on its complexity. Indeed, although according to the Seeking-Experiencing Divine Forgiveness Model the process of seeking God’s forgiveness after committing a sin begins as soon as a person decides to seek this form of forgiveness after wrongdoing (Fincham and Maranges, 2024), our findings show that we cannot conceptualize divine forgiveness as wholly unconditional. Indeed, for one-third of the sample, it depends on specific conditions, which are not entirely equivalent across monotheistic religions. Also, about 11.8% of the sample indicated that ‘God never fully forgives’, an interesting finding because one may argue that God’s forgiveness should be complete (see, for example, Fincham, 2022; Kim and Enright, 2014), but some believers offer different and subjective viewpoints - maybe under various influences, such as one’s God image, one’s religious commitment, or one’s conception of forgiveness in general.

Our findings suggest that existing models and measures of divine forgiveness could be refined by incorporating religious affiliation as a factor that shapes both the conceptualization and operationalization of sin and the strategies individuals use to seek forgiveness. Models and related measures that ignore religious context risk missing meaningful variation in how individuals understand and pursue forgiveness. In this light, beyond merely identifying differences between groups, our findings suggest the need for theoretical frameworks of divine forgiveness to adopt a more contextually sensitive perspective. Here, the complexity of divine forgiveness becomes evident in its very nature.

This study represents a significant and fundamental step forward in the study of divine forgiveness. Taken together, analyzing the conceptualization of divine forgiveness from diverse religious perspectives can help identify both similarities and differences, allowing these insights to be integrated at theoretical, psychometric, and intervention levels. Moreover, such an analysis could foster a more meaningful dialogue and exchange between different religions by enhancing awareness of both shared elements and unique aspects within each tradition. Accordingly, interreligious dialogue may benefit from this knowledge. The knowledge acquired through both the focus group and the questionnaire is highly relevant for developing and validating a self-report scale to measure dispositional divine forgiveness both within and across the three main monotheistic religions considered here. Indeed, the existing measures are too brief and simplistic to capture the complexity of the construct, resulting in poor content validity, and have not been adequately validated (Fincham and May, 2019; Harris et al., 2008; Kim et al., 2022; Toussaint et al., 2001). While it is important for a scale to be parsimonious to improve participation rates, minimize participants’ burden and fatigue, reduce the risk of careless response bias, and save assessment time and related costs (Kemper et al., 2019; Ward and Meade, 2018), it is equally essential to use a measure having high content validity across religions to generate more robust and scientifically grounded knowledge on its assessment and effects.

Finally, the findings of the present study also have relevant practical implications. In particular, we argue that comprehending the meaning ascribed to divine forgiveness may be particularly helpful in clinical and spiritual interventions. Understanding, seeking and reaching divine forgiveness after a sin committed for lay believers may allow people to better deal with pain and conflicts in a positive and constructive way. Indeed, divine forgiveness may allow a positive inner transformation and the achievement of a sense of peace. Accordingly, a meta-analysis in this field has highlighted that religious/spiritual interventions may reduce pathological symptoms, especially anxiety (Gonçalves et al., 2015). Further knowledge on the multidimensional nature of divine forgiveness and the conditions that influence it may help clinicians better understand this complex phenomenon among believers and aid in developing a more comprehensive way of working with people who, in clinical settings, feel the need to undertake the process of seeking divine forgiveness after committing a transgression or sin.

## Data Availability

The datasets presented in this study can be found in online repositories. The names of the repository/repositories and accession number(s) can be found at: Open Science Framework at https://osf.io/8tkh2/.

## Footnotes

^1^16.5% of respondents did not specify any religious confession when directly asked. Almost all Christian participants were Catholic (29.4% of the entire sample), Jews were Ashkenazi (15.7% of the entire sample) and Sephardic (6.8% of the entire sample). Finally, almost all Muslims respondents (20.3% of the entire sample) reported to be Sunni.

^2^We had originally planned to collect all these self-report data in Italy, but the main Jewish communities we contacted in Italy declined to participate in the research for fear of exposing their members to higher risks of identification and reprisals, due to the Palestine-Israel war, which had made Italian Jews more vulnerable to threats and attacks. Data from Christians and Muslims were therefore collected in Italy through convenience sampling by involving students and asking them to distribute the questionnaire among their acquaintances. We also shared the questionnaire through social media (Telegram, Facebook, Instagram, WhatsApp groups of students and/or prayer groups). Data from Jews were primarily collected in Israel, among English-speaking believers, through Prolific.

^3^We combined “harm to self” and “harm to others” into a single category because many responses were vague — for example, simply stating “doing harm” — or included both.

^4^When conducting the binomial logistic regression models without accounting for the included covariates, two differences emerge compared to the results presented here. Specifically, no significant differences were found between Jews and Christians with regard to the word Compassion (Wald = 3.15, *p* = 0.076), and no significant differences were found with regard to the word Sin [*χ*^2^(2) = 5.49; *p* = 0.064; Nagelkerke *R*^2^ = 0.07; cases correctly classified = 95.2%], also according to the non-significant Wald tests.

^5^When conducting the binomial logistic regression models without accounting for the included covariates no differences emerge compared to the results presented here.

^6^When conducting the binomial logistic regression models without accounting for the included covariates, the pattern of results remained the same, with two exceptions. Specifically, when the covariates were not included no significant differences were found between Jews and Muslims with regard to the condition sincere repentance (Wald = 2.69, *p* = 0.101), and no significant differences were found with regard to the condition committing to not repeat the wrongdoing among any of the groups considered [*χ*^2^(2) = 4.06; *p* = 0.132; Nagelkerke *R*^2^ = 0.05; cases correctly classified = 93.9%].

## References

[ref1] Aggarwal S. Wright J. Morgan A. Patton G. Reavley N. (2023). Religiosity and spirituality in the prevention and management of depression and anxiety in young people: a systematic review and meta-analysis. BMC Psychiatry 23:729. doi: 10.1186/s12888-023-05091-2, PMID: 37817143 PMC10563335

[ref2] Akl M. Mullet E. (2010). Forgivingness: relationships with conceptualizations of divine forgiveness and childhood memories. Int. J. Psychol. Relig. 20, 187–200. doi: 10.1080/10508619.2010.481226

[ref3] Bartholomaeus J. Mandrell J. Strelan P. (2025). A prototype analysis of divine forgiveness. Psychol. Relig. Spirit. 2:555. doi: 10.1037/rel0000555

[ref4] Bassett R. L. Carrier E. Charleson K. Pak N. R. Schwingel R. Majors A. . (2016). Is it really more blessed to give than to receive? A consideration of forgiveness and perceived health. J. Psychol. Theol. 44, 28–41. doi: 10.1177/009164711604400103

[ref5] Böttigheimer C. Kamp K. (2025). The concept of sin in Judaism, Christianity and Islam. Boston: De Gruyter.

[ref6] Bowen G. A. (2005). Preparing a qualitative research-based dissertation: lessons learned. Qual. Rep. 10, 208–222. doi: 10.46743/2160-3715/2005.1846

[ref7] Braam A. W. Koenig H. G. (2019). Religion, spirituality and depression in prospective studies: a systematic review. J. Affect. Disord. 257, 428–438. doi: 10.1016/j.jad.2019.06.063, PMID: 31326688

[ref8] Carpenter T. P. Carlisle R. D. Tsang J. A. (2014). Tipping the scales: conciliatory behavior and the morality of self-forgiveness. J. Posit. Psychol. 9, 389–401. doi: 10.1080/17439760.2014.910823

[ref9] Chen Y. Harris S. K. Worthington E. L. Jr. VanderWeele T. J. (2019). Religiously or spiritually-motivated forgiveness and subsequent health and well-being among young adults: an outcome-wide analysis. J. Posit. Psychol. 14, 649–658. doi: 10.1080/17439760.2018.1519591, PMID: 31360213 PMC6662928

[ref10] Coelho-Júnior H. J. Calvani R. Panza F. Allegri R. F. Picca A. Marzetti E. . (2022). Religiosity/spirituality and mental health in older adults: a systematic review and meta-analysis of observational studies. Front. Med. 9:877213. doi: 10.3389/fmed.2022.877213, PMID: 35646998 PMC9133607

[ref11] DeBono A. Shariff A. F. Poole S. Muraven M. (2017). Forgive us our trespasses: priming a forgiving (but not a punishing) god increases unethical behavior. Psychol. Relig. Spiritual. 9, S1–S10. doi: 10.1037/rel0000105, PMID: 40991787

[ref12] Dorff E. N. (1998). “The elements of forgiveness: a Jewish approach” in Dimensions of forgiveness: psychological research and theological perspectives. ed. Worthington E. L. (London: Templeton Foundation), 29–55.

[ref13] Fincham F. D. (2022). Towards a psychology of divine forgiveness. Psychol. Relig. Spirit. 14, 451–461. doi: 10.1037/rel0000323

[ref14] Fincham F. D. Maranges H. M. (2024). Psychological perspectives on divine forgiveness: seeking divine forgiveness. Front. Psychol. 15:1256402. doi: 10.3389/fpsyg.2024.1256402, PMID: 38455121 PMC10918850

[ref15] Fincham F. D. Maranges H. M. (2025). Psychological perspectives on divine forgiveness: 2. Does viewing god as intervening account for the association between god image and divine forgiveness? Psychol. Relig. Spirit. 17, 65–70. doi: 10.1037/rel0000531

[ref16] Fincham F. D. May R. W. (2019). Self-forgiveness and well-being: does divine forgiveness matter? J. Posit. Psychol. 14, 854–859. doi: 10.1080/17439760.2019.1579361

[ref17] Fincham F. D. May R. (2024). Divine forgiveness and well-being among emerging adults in the USA. J. Relig. Health 63, 2276–2290. doi: 10.1007/s10943-022-01678-3, PMID: 36183033

[ref18] Flannelly K. (2017). Religious beliefs, evolutionary psychiatry, and mental health in America: Evolutionary threat assessment systems theory. Cham: Springer.

[ref19] Garssen B. Visser A. Pool G. (2021). Does spirituality or religion positively affect mental health? Meta-analysis of longitudinal studies. Int. J. Psychol. Relig. 31, 4–20. doi: 10.1080/10508619.2020.1729570

[ref20] Gonçalves J. P. B. Lucchetti G. Menezes P. R. Vallada H. (2015). Religious and spiritual interventions in mental health care: a systematic review and meta-analysis of randomized controlled clinical trials. Psychol. Med. 45, 2937–2949. doi: 10.1017/S0033291715001166, PMID: 26200715 PMC4595860

[ref21] Harris S. K. Sherritt L. R. Holder D. W. Kulig J. Shrier L. A. Knight J. R. (2008). Reliability and validity of the brief multidimensional measure of religiousness/spirituality among adolescents. J. Relig. Health 47, 438–457. doi: 10.1007/s10943-007-9154-x, PMID: 19093673

[ref22] Huber S. Allemand M. Huber O. W. (2011). Forgiveness by god and human forgivingness: the centrality of the religiosity makes the difference. Arch. Psychol. Relig. 33, 115–134. doi: 10.1163/157361211X565737

[ref23] James A. Wells A. (2003). Religion and mental health: towards a cognitive-behavioural framework. Br. J. Health Psychol. 8, 359–376. doi: 10.1348/135910703322370905, PMID: 14606978

[ref24] Kearns J. N. Fincham F. D. (2004). A prototype analysis of forgiveness. Personal. Soc. Psychol. Bull. 30, 838–855. doi: 10.1177/0146167204264237, PMID: 15200691

[ref25] Kelliher Rabon J. Webb J. R. Chang E. C. Hirsch J. K. (2018). Forgiveness and suicidal behavior in primary care: mediating role of future orientation. J. Spiritual. Ment. Health 21, 1–13. doi: 10.1080/19349637.2018.1469454

[ref26] Kemper N. S. Campbell D. S. Earleywine M. Newheiser A. K. (2019). Likert, slider, or text? Reassurances about response format effects. Addict. Res. Theory 28, 406–414. doi: 10.1080/16066359.2019.1676892

[ref27] Kim J. J. Enright R. D. (2014). Differing views on forgiveness within Christianity: do graduate-level theology students perceive divine and human forgiveness differently? Spiritual. Clin. Pract. 1, 191–202. doi: 10.1037/scp0000027

[ref28] Kim J. Ragasajo L. J. C. Kolacz R. L. Painter K. J. Pritchard J. D. Wrobleski A. (2022). The interplay between divine, victim, and self-forgiveness: the relationship between three types of forgiveness and psychological outcomes. J. Psychol. Theol. 50, 414–427. doi: 10.1177/00916471211046226

[ref29] Krause N. Ellison C. G. (2003). Forgiveness by god, forgiveness of others, and psychological well-being in late life. J. Sci. Study Relig. 42, 77–93. doi: 10.1111/1468-5906.00162, PMID: 21373377 PMC3046863

[ref30] Krause N. Ironson G. (2017). Forgiveness by god, religious commitment, and waist/hip ratios. J. Appl. Biobehav. Res. 22:4. doi: 10.1111/jabr.12104

[ref31] Lambert N. M. Fincham F. D. Braithwaite S. R. Graham S. M. Beach S. R. H. (2009). Can prayer increase gratitude? Psychol. Relig. Spirit. 1, 139–149. doi: 10.1037/a0016731

[ref32] Long K. N. Chen Y. Potts M. Hanson J. VanderWeele T. J. (2020). Spiritually motivated self-forgiveness and divine forgiveness, and subsequent health and well-being among middle-aged female nurses: an outcome-wide longitudinal approach. Front. Psychol. 11:1337. doi: 10.3389/fpsyg.2020.01337, PMID: 32733311 PMC7363844

[ref33] Ludwig J. M. Koetke J. Schumann K. (2025). Implications of divine forgiveness for conciliatory behavior: understanding how feeling forgiven by god influences apologies via self-forgiveness, gratitude, and humility. Personal. Soc. Psychol. Bull. 12:1461672241312265. doi: 10.1177/01461672241312265, PMID: 39936562

[ref34] Maltby J. Day L. (2000). Depressive symptoms and religious orientation: examining the relationship between religiosity and depression within the context of other correlates of depression. Pers. Individ. Differ. 28, 383–393. doi: 10.1016/S0191-8869(99)00108-7

[ref35] Maltby J. Lewis C. A. Day L. (1999). Religious orientation and psychological well-being: the role of the frequency of personal prayer. Br. J. Health Psychol. 4, 363–378. doi: 10.1348/135910799168704

[ref36] McConnell J. M. Dixon D. N. (2012). Perceived forgiveness from god and self-forgiveness. J. Psychol. Christ. 31, 31–39.

[ref37] McCullough M. E. (2000). Forgiveness as human strength: theory, measurement, and links to well-being. J. Soc. Clin. Psychol. 19, 43–55. doi: 10.1521/jscp.2000.19.1.43

[ref38] McCullough M. E. Pargament K. I. Thoresen C. E. (2000). Forgiveness: Theory, research, and practice. New York: The Guilford Press.

[ref39] Moucarry C. G. (2004). The search for forgiveness: Pardon and punishment in Islam and Christianity. Illinois: Inter-Varsity Press.

[ref40] Mullet E. Azar F. (2009). Apologies, repentance, and forgiveness: a Muslim–Christian comparison. Int. J. Psychol. Relig. 19, 275–285. doi: 10.1080/10508610903146274

[ref41] Paleari F. G. Donato S. Iafrate R. Regalia C. (2009). “The tendency to forgive in premarital couples: reciprocating the partner or reproducing parental dispositions?” in Psychology of relationships. eds. Cuyler E. Ackhart M. (New York: Nova Science Publishers).

[ref42] Pargament K. (1990). God help me: toward a theoretical framework of coping for the psychology of religion. Res. Soc. Sci. Study Relig. 2, 195–224. doi: 10.1007/BF00938065

[ref43] Pargament K. I. (1996). “Religious methods of coping: resources for the conservation and transformation of significance” in Religion and the clinical practice of psychology. ed. Shafranske E. (New York: APA Books), 2l5–2l239.

[ref44] Pelucchi S. Regalia C. Paleari G. Fincham F. D. (2017). “Self-forgiveness within couple transgressions” in Handbook of the psychology of self-forgiveness. eds. Woodyatt L. Worthington E. L. Jr. Wenzel M. Griffin B. J. (Cham: Springer), 115–130.

[ref45] Peters F. (2018). The children of Abraham: Judaism, Christianity, Islam. Princeton: Princeton University Press.

[ref46] Pew-Templeton Global Religious Futures project (2022). Key findings from the global religious futures project. Available online at: https://www.pewresearch.org/religion/2022/12/21/key-findings-from-the-global-religious-futures-project/

[ref47] Poloma M. M. Gallup G. H. (1991). Varieties of prayer: a survey report. Norcross, GA: Trinity Press.

[ref48] Rye M. S. Pargament K. I. Ali M. A. Beck G. L. Dorff E. N. Hallisey C. . (2000). “Religious perspectives on forgiveness” in Forgiveness: theory, research, and practice. eds. McCullough M. E. Pargament K. I. Thoresen C. E. (New York, NY: Guilford Press), 17–40.

[ref49] Saleem S. Sitwat A. (2025). Understanding the dynamics of divine forgiveness to resolve interpersonal transgressions: an Islamic perspective. Pak. J. Humanit. Soc. Sci. 13, 21–31. doi: 10.52131/pjhss.2025.v13i1.2630

[ref50] Sharp C. A. Davis E. B. George K. Cuthbert A. D. Zahl B. P. Davis D. E. . (2021). Measures of god representations: theoretical framework and critical review. Psychol. Relig. Spirit. 13, 340–357. doi: 10.1037/rel0000257

[ref51] Silton N. R. Flannelly K. J. Galek K. Ellison C. G. (2014). Beliefs about god and mental health among American adults. J. Relig. Health 53, 1285–1296. doi: 10.1007/s10943-013-9712-3, PMID: 23572240

[ref52] Skalski-Bednarz S. B. Webb J. R. Wilson C. M. Toussaint L. L. Surzykiewicz J. Reid S. D. . (2024). Pathways to flourishing: the roles of self- and divine forgiveness in mitigating the adverse effects of stress and substance use among adults in Trinidad and Tobago. Religion 15:1060. doi: 10.3390/rel15091060

[ref53] Statista (2022). Share of global population affiliated with major religious groups in 2022, by religion. Available online at: https://www.statista.com/statistics/374704/share-of-global-population-by-religion/

[ref54] Stulp H. P. Koelen J. Schep-Akkerman A. Glas G. G. Eurelings-Bontekoe L. (2019). God representations and aspects of psychological functioning: a meta-analysis. Cogent Psychol. 6:1647926. doi: 10.1080/23311908.2019.1647926

[ref55] Subkoviak M. J. Enright R. D. Wu C. R. Gassin E. A. Freedman S. Olson L. M. . (1995). Measuring interpersonal forgiveness in late adolescence and middle adulthood. J. Adolesc. 18, 641–655. doi: 10.1006/jado.1995.1045

[ref56] Svalina S. S. Webb J. R. (2012). Forgiveness and health among people in outpatient physical therapy. Disabil. Rehabil. 34, 383–392. doi: 10.3109/09638288.2011.607216, PMID: 22017571

[ref57] Toussaint L. L. Owen A. D. Cheadle A. (2012). Forgive to live: forgiveness, health, and longevity. J. Behav. Med. 35, 375–386. doi: 10.1007/s10865-011-9362-4, PMID: 21706213

[ref58] Toussaint L. L. Webb J. R. Hirsch J. K. (2017). “Self-forgiveness and health: a stress-and-coping model” in Handbook of the psychology of self-forgiveness. eds. Woodyat L. Worthington J. Wenzel M. Griffin B. J. (Cham: Springer), 87–99.

[ref59] Toussaint L. L. Williams D. R. Musick M. A. Everson S. A. (2001). Forgiveness and health: age differences in a U.S. probability sample. J. Adult Dev. 8, 249–257. doi: 10.1023/a:1011394629736

[ref60] Tsang J. A. McCullough M. E. Hoyt W. T. (2005). Psychometric and rationalization accounts of the religion-forgiveness discrepancy. J. Soc. Issues 61, 785–805. doi: 10.1111/j.1540-4560.2005.00432.x

[ref61] Upenieks L. Ellison C. G. Krause N. M. (2023). “To err is human, to forgive, divine”: religious doubt, psychological well-being and the moderating role of divine forgiveness. J. Relig. Spiritual. Aging 37, 98–119. doi: 10.1080/15528030.2023.2262406, PMID: 39936161 PMC11810121

[ref62] Van Oyen Witvliet C. (2001). Forgiveness and health: review and reflections on a matter of faith, feelings, and physiology. J. Psychol. Theol. 29, 212–224. doi: 10.1177/009164710102900303

[ref63] Ward M. K. Meade A. W. (2018). Applying social psychology to prevent careless responding during online surveys. Appl. Psychol. 67, 231–263. doi: 10.1111/apps.12118

[ref64] Woodyatt L. (2017). Handbook of the psychology of self-forgiveness. Cham: Springer.

[ref65] Woodyatt L. Wenzel M. (2013). Self-forgiveness and restoration of an offender following an interpersonal transgression. J. Soc. Clin. Psychol. 32, 225–259. doi: 10.1521/jscp.2013.32.2.225

[ref66] Worthington E. L. Wade N. G. (2019). Handbook of forgiveness. New York: Routledge.

[ref67] Yaden D. B. Batz-Barbarich C. L. Ng V. Vaziri H. Gladstone J. N. Pawelski J. O. . (2022). A meta-analysis of religion/spirituality and life satisfaction. J. Happiness Stud. 23, 4147–4163. doi: 10.1007/s10902-022-00558-7

