# Supplementary material for: Core facets of divine forgiveness: a study across monotheistic religions
Source: Front Psychol. 2025 Nov 6;16:1646554. doi: 10.3389/fpsyg.2025.1646554 (PMC12631403; doi:10.3389/fpsyg.2025.1646554)
Supplement: Supplementary file 1 [file Supplementary_file_1.docx]

Appendix 1.

Focus Group Guide - Questions posed to answer the research aims

Part One - Introduction: Divine forgiveness in the Christian/Jewish/Muslim religion

*In your religion/according to your discipline's perspective:*

1. *What is divine forgiveness? Is there a shared definition of divine forgiveness?*
2. *What is the relationship between God and forgiveness?*
3. *What are the key words of divine forgiveness?*
4. *Does forgiveness coincide with mercy or are there differences between them? (Which ones?)*

Part Two - The experience of forgiveness: Sin

1. *What is meant by the term "sin?*
2. *Are there differences between sin against God and sin against other human beings? (What are the main ones?)*

Part Three - The experience of forgiveness: Conditions

1. *What are the conditions - in terms of thoughts, actions, and feelings - for being forgiven by God?*

Appendix 2

Part a) socio demographic questions;

Part b) ad hoc questions aimed at measuring the religious involvement;

- *Are you a believer? Yes/No;*
- *If yes, how much would you define yourself as a believer? (from 1 = Little, to 4 = Very Much);*
- *In which of the following religions do you identify? Christianity/Judaism/Islam/Other;*
- *How often do you pray or participate in group religious rituals?(from 1 = Never, to 6 = Every day);*
- *How well do you know the doctrine (principles and teachings) of your religion? (from 1 = Not at all, to 4 = Very Much);
  How much do you agree with the doctrine (principles and teachings) of your religion? (from 1 = Not at all, to 4 = Very Much).*

Part c) ad hoc questions aimed at assessing what divine forgiveness is;

- *List 3 keywords that come to mind when thinking about divine forgiveness:*

Part d) ad hoc questions aimed at assessing what sins are;

- *For what types of sin, in your opinion, should one ask forgiveness from God? For sins against God himself/For sins against other people/For sins against creation or nature/ For all types of sin.*
- *In your opinion, what is a sin?______________*

Part e) which are the conditions under which divine forgiveness occurs

- *In your opinion: 1)* *God forgives only if (specify under what conditions or circumstances God forgives): _________________* 2) *God forgives always, unconditionally* 3) *God never forgives completely and constantly puts us to the test*
- *Express your level of agreement/disagreement with the following statements:*

*a. God forgives me when I commit serious sins (from 1 = Disagree, to 5 = Agree);*

*b. God forgives me when I commit minor sins (from 1 = Disagree, to 5 = Agree);*

*c. God forgives me when I commit offenses (from 1 = Disagree, to 5 = Agree);*

*d. God forgives me when I do something wrong (from 1 = Disagree, to 5 = Agree).*
